# Usability and Acceptance by Therapists and Users of an Internet‐Based Intervention Based on the Unified Protocol in Argentina

**DOI:** 10.1002/jclp.70037

**Published:** 2025-08-20

**Authors:** Milagros Celleri, Florencia Klajner, F. Camila Cremades, Cristian Javier Garay, Martin Etchevers, Jorge Osma

**Affiliations:** ^1^ Universidad de Buenos Aires, Facultad de Psicología, Secretaría de Investigaciones, Ciudad Autónoma de Buenos Aires Buenos Aires Argentina; ^2^ Facultad de Psicología y Sociología Universidad de Zaragoza Teruel Spain; ^3^ Instituto de Investigación Sanitaria de Aragón Zaragoza Spain

**Keywords:** acceptability, emotional disorders, internet‐based intervention, unified protocol, usability

## Abstract

Latin American countries face a significant mental health gap, characterized by an increasing prevalence of mental disorders and limited access to evidence‐based treatments. Internet‐based interventions hold promise for bridging this gap. In Argentina, there are currently no locally developed internet‐based interventions. To address this gap, our research team recently developed an Internet‐Based Unified Protocol Intervention (IUPI), a transdiagnostic intervention adapted from the Unified Protocol. This study aimed to evaluate its usability and acceptability in the local population, a necessary step for its subsequent implementation. Methodology: A mixed‐methods study was conducted with 20 professionals and 10 users who tested IUPI for 2 weeks. The System Usability Scale, an ad‐hoc questionnaire, and focus groups were conducted. Results: Usability scores indicated good usability for both professionals (*M* = 71.37 SD = 19.61) and users (*M* = 73.75 SD = 2.60). Both groups expressed willingness to use and recommend the platform. Thematic analysis revealed the perception that these interventions can enhance access to treatment. On the other hand, barriers such as connectivity issues, limited resources, and perceived lack of warmth and support were described. Discussion: This is the first usability study of an internet‐based intervention in Argentina. Findings are promising for cultural adaptation and broader implementation, potentially increasing access to psychotherapeutic treatments.

## 1 Introduction

Anxiety, depression, and related disorders, called Emotional Disorders (EDs) because of their shared etiological and maintenance mechanisms (Bullis et al. 2019), are the most prevalent disorders worldwide (Pan American Health Organization 2018; World Health Organization 2022). In Argentina, they reach a lifetime prevalence of 16.4% and 8.7% respectively (Cia et al. 2018; Stagnaro et al. 2018). While there are well‐positioned evidence‐based treatments for addressing these psychopathologies, 76%–85% of the population in Low‐ and Middle‐Income Countries (LMICs) do not access these evidence‐based treatments (Araya et al. 2018; Pan American Health Organization 2018; Pérez‐Padilla et al. 2017). LMICs face the mental health gap, an increase in mental health problems, and limited access to health services (Alloh et al. 2018; Arjadi et al. 2015; Patel 2007). In addition, the availability of health services is limited, making access to treatment even more difficult (Eaton et al. 2011). In Argentina, the population's difficulties in accessing psychotherapeutic treatment have increased due to a lack of medical coverage or availability in health centers (Etchevers et al. 2021). In turn, the availability of therapists trained in evidence‐based therapies is scarce (Klinar et al. 2019). Moreover, this gap in access to mental health services in Argentina may be further exacerbated by the digital gap (Pan American Health Organization 2016). In rural areas, only 52% of the population has internet access, and in some locations, connectivity is intermittent (Instituto Nacional de Estadística y Censo INDEC 2022). In contrast, eight out of 10 people own a mobile phone, and there is more than one mobile internet subscription per capita (World Health Organization, WHO 2016).

Internet‐based Interventions are cognitive‐behavioral interventions (ICBT) adapted to be delivered through digital platforms (Andersson 2024; Molloy et al. 2021). ICBT can potentially increase the dissemination of treatments and reduce the access gap to health systems (Andersson 2024), which are less costly and potentially more efficient (Andersson and Titov 2014; Christensen 2010). They have been studied mainly in high‐income countries (Andersson and Cuijpers 2009), where they are effective in the treatment of a wide range of disorders (Etzelmueller et al. 2020). In contrast, in Latin America, the evidence is limited, and the treatment access gap is higher, especially in low socioeconomic groups (Andersson and Titov 2014). At the moment, studies conducted in the region mainly address depression and substance use, and only three controlled studies have been conducted (Arjadi et al. 2015; Jiménez‐Molina et al. 2019) with modest results.

On the other hand, given the high comorbidity among EDs (Bullis et al. 2019) and the large proliferation of specific protocols in the field of Cognitive Behavioral Therapies (CBT) (Hofmann and Hayes 2024), transdiagnostic approaches have recently been developed that allow for a more cost‐effective approach (Mansell et al. 2009). Along these lines, there is currently a wide range of interventions based on emotional regulation that have been shown to be effective (Saccaro et al. 2024), one of which, the Unified Protocol (UP), was developed by David Barlow et al. (2018). The UP is a transdiagnostic CBT for ED, focused on improving emotional regulation strategies (Farchione et al. 2024). It is as effective as specific protocols for both individual and group applications (Ayuso‐Bartol et al. 2024; Bullis et al. 2023; Peris‐Baquero and Osma 2023) and has also begun to be studied in an internet‐based intervention format (Eustis et al. 2024; Schaeuffele et al. 2022).

One of the barriers reported in previous literature for the implementation of ICBT is that many of them have not been tested for usability (Dugstad Wake et al. 2024; Stoddard et al. 2006). Assessing the usability and acceptability of ICBT is crucial. While there are various definitions of usability, in general terms, it refers to the ability of a system to be used by end users in a satisfactory, easy, and effective way (Lewis and Sauro 2021; Sauer et al. 2020). In this process, it is essential to incorporate end‐user feedback (Harst et al. 2021), as usability is a prerequisite and predictor for the further dissemination and implementation (Harst et al. 2019). Such studies allow for making the necessary changes and redefining the intervention according to the perspective and needs of the end‐users (Skivington et al. 2021).

So far, no ICBT specifically designed for our population has been developed or tested in Argentina. Therefore, we developed during the years 2021–2023 IUPI *“Internet‐Based Unified Protocol Intervention*” (Celleri et al. 2022), an internet‐based intervention of cognitive behavioral character based on the UP, jointly between researchers from the Faculty of Psychology and the Faculty of Engineering at the University of Buenos Aires.

The present study aims to evaluate the usability and acceptability of the first version of IUPI (Celleri et al. 2021) among the Argentine population, including both mental health professionals and users.

## 2 Method

### 2.1 Desing

A mixed‐design, exploratory, descriptive, and cross‐sectional study was conducted (Kazdin 2021). A mixed method has been chosen as it has been proposed as the recommended method for the development of health technology applications (Maramba et al. 2019). In the discussion, an integration between the quantitative and the qualitative results obtained will be made. The guidelines for Good Reporting of A Mixed Methods Study (Supporting Information S1: Appendix 1) were followed (GRAMMS; O'cathain et al. 2008; Schoonenboom and Johnson 2017).

### 2.2 Participants

A sample of 20 professionals and 10 users participated in this usability study. The sample was collected on a purposive, non‐probabilistic basis. To determine the sample size, we followed the guidelines proposed by Hwang and Salvendy (2010) for usability studies. The therapists were recruited via contact among colleagues, public hospitals, and dissemination via the research team's social networks. To ensure sample diversity, participants both familiar and unfamiliar with the Unified Protocol were included to capture a range of clinical perspectives. Professionals from both the public and private sectors were recruited to gather insights into implementation across different settings. In addition, therapists from Buenos Aires and provinces in the interior of the country were included to reflect the realities of regions with the greatest digital divide. The users included former participants of an ongoing study of the group application of the UP (Celleri et al. 2023) who were familiar with the protocol, and the general public, prioritizing people who reported anxious or depressive symptoms. Table 1 shows the characteristics of the sample.

**TABLE 1:** Sociodemographic characteristics of users and professionals.

Variable	Users (*n* = 10)			Professionals (*n* = 20)		
	*n*	%	Mean (*ED*)	*n*	%	Mean (*ED*)
Age			33.80 (9.41)			34.15 (7.46)
Gender						
Male	0	0		4	20	
Female	10	100		16	80	
Residence						
CABA	3	30		6	30	
AMBA	5	50		6	30	
Buenos Aires	2	20		3	15	
Countryside	0	0		5	25	
Educational level						
High school	1	10		0	0	
College	8	80		14	70	
Posgraduate	1	10		6	30	
Prior knowledge of internet‐based interventions						
No	7	70		7	35	
Yes	3	30		12	60	
Do not know	0	0		1	5	
Prior knowledge of the Unified Protocol						
No	7	70		4	20	
Yes	3	30		16	80	
Background in psychotherapy						
Yes	8	80		—		
No	2	20		—		
Field of work						
Public sector	—			6	30	
Private sector	—			14	70	

Abbreviations: AMBA, Buenos Aires Metropolitan Area; CABA, Autonomous City of Buenos Aires.

Inclusion criteria for users were: (1) being over 18 years old, (2) accepting informed consent, (3) residing in Argentina, and (4) reporting emotional symptoms or having previously participated in the research team's therapeutic groups.

For the group of therapists, the criteria were (1) to accept the informed consent, (2) to reside in Argentina, (3) to have a valid registration from the place of care, and (4) to have training in cognitive‐behavioral therapies. It was not a requirement to have previous knowledge of the UP.

Of the 20 therapists and 10 users who participated by completing the form after using IUPI, 16 therapists and six users participated in the focus groups. The rest of the participants did not respond to the calls and e‐mails to participate, so it was not possible to include them.

### 2.3 IUPI Beta Version

The intervention program IUPI beta version was developed in an interdisciplinary way between the Faculty of Psychology and the Faculty of Engineering at the University of Buenos Aires, during the years 2021, 2022, and 2023, in the context of a strategic development project funded by the Secretariat of Science and Technology of the University of Buenos Aires.

Four researchers, psychologists and psychiatrists, from our team participated in the development and cultural adaptation of the UP to the content of the platform (videos, animations, texts, and audios). The development of the web platform was carried out by a team of engineers and students of systems engineering in systems from the Advanced Information Systems Laboratory (LSIA). Technical details on the technologies used in the development of IUPI can be found at (Celleri et al. 2022).

First, to begin with the development of IUPI and the adaptation of the UP (Barlow et al. 2018), permission was obtained from its main author, David Barlow, via email. Once permission was obtained, work began on adapting and recording the different modules to the characteristics and specific examples of the problems most associated with our population (Korman et al. 2022; Korman and Sarudiansky 2023). The content of each of the modules, as well as examples of the exercises included, are presented in Supporting Information S1: Appendix 2. To exemplify some of the concepts, two characters have been created: Carla and Juan, who, through animations, show how to complete some of the records and exemplify concepts.

IUPI has two main modes: therapist mode and patient mode. The functions of each of the accesses are described in Celleri et al. (2022).

At the beginning of each module, the Overall Anxiety Severity and Impairment Scale (OASIS; Norman et al. 2006) and Overall Depression Severity and Impairment Scale (ODSIS; Bentley et al. 2014) which were previously adapted and validated to our population by the research team (Rojas et al. 2023), was provided to be able to measure the evolution of each participant weekly. Once completed, the platform displays a time series graph that allows the user to see their progress.

The intervention is divided into 9 weeks, each corresponding to a module of the UP. At the same time, an initial session is included, which we call module 0, where the intervention is presented to the therapists, how to complete the OASIS and ODSIS scales, and the functional model of the EDs.

Although for the usability test participants were given full access to all modules, the platform was designed to operate sequentially, that is, the user has 1 week to complete the exercises to advance to the next module. In case of failure to complete, the platform locks and the therapist must reassign access.

### 2.4 Procedure

Participants were recruited through outreach to colleagues, Faculty of Psychology staff, and flyers on social media. Former participants of therapeutic groups coordinated by the team were contacted via email. The test was carried out during the period from May to October 2024. Those interested in participating received information by email about the requirements and objectives to be able to participate in the study.

Once the informed consent was signed, participants were sent the platform access data (username, password, and link) via email, with instructions on how long they had to use the platform. Participants used IUPI for 2 weeks. Therapists were granted access to both the patient and therapist user profiles, while patients were granted access only to the patient profile. During the 2‐week period, participants were instructed to explore the platform, view the videos and animations, complete the assessment scales, and fill out the monitoring forms.

Once the trial period was over, they were invited to participate in the focus group and were sent an online form to complete the questionnaires and the usability scale. The focus groups were conducted virtually to allow individuals from all over the country to participate.

They were coordinated by two researchers from the team (MC & KF) on each occasion, following the guidelines proposed by Gundumogula (2020). Each focus group included an average of six participants and lasted on average 1.5 h. Permission was obtained from the participants to record the focus groups, which were then recorded for data analysis. For ethical reasons, once the analysis of the interviews was completed, the original audio recordings were deleted. Details of the focus group procedure and analysis are given in Supporting Information S1: Appendix 3.

### 2.5 Éthics

All study participants received and signed an informed consent form in which the voluntary nature of their participation was made explicit, as well as their right to leave the study at any stage. The consent was framed according to local legislation in accordance with the Patients' Rights Act and the Declaration of Helsinki.

The present study obtained the approval of the Responsible Conduct Committee of the Faculty of Psychology at the University of Buenos Aires and was framed within an accredited UBACYT project funded by the Faculty of Psychology at the University of Buenos Aires.

### 2.6 Instruments

#### 2.6.1 Ad‐Hoc Socio‐Demographic Questionnaire

An ad‐hoc socio‐demographic questionnaire was constructed among the members of the research team and administered to all participants. It collected information on gender, place of residence, age, and the highest level of education attained. Information on prior knowledge of internet‐based interventions and the Unified Protocol was also collected. In the case of patients, background information on psychotherapy was collected, while for therapists, information was collected on their clinical work setting (public/private).

#### 2.6.2 Ad‐Hoc Satisfaction Questionnaire

To assess the level of liking and clarity regarding the esthetics and design, level of comprehension, and information offered on the platform, a satisfaction questionnaire was constructed based on previous studies (Bureau et al. 2021). The Likert‐type response items consisted of five items. Regarding how user‐friendly they found the application interface, the score was determined as 1 not very user‐friendly to 5 very user‐friendly; while, for clarity, the response options were indicated as 1 unclear to 5 very clearly. In addition, questions were included that probed the willingness to use the platform as an adjunct to therapy and to recommend the use of the platform to others.

#### 2.6.3 System Usability Scale (SUS; Brooke 1996; Spanish Adaptation by Sevilla‐Gonzalez et al. 2020)

This is a widely used scale to assess the usability of systems, including those platforms developed for eHealth interventions (Pan American Health Organization 2016) such as internet‐based interventions (Rahmadiana et al. 2021). The version translated and validated in Spanish (Castilla et al. 2023) was used. The instrument consists of 10 items with statements regarding the experience of using the platform and five Likert response options according to the degree of agreement with these statements; 1 being totally disagree and 5 totally agree. The items are presented in alternating order of positive statements about the platform (odd items) and negative statements about the platform (even items) to avoid participant response bias. It has good psychometric properties (Cronbach's α = 0.812). Scores are divided into not acceptable (0–50), marginal (50–70) and acceptable (70–100). Within acceptable, scores are divided into good (70–80), excellent (80–90), and best imaginable (90–100) (Bangor et al. 2009).

#### 2.6.4 Semi‐Structured Interviews

Two semi‐structured interviews were designed: one consisting of 16 questions in the case of the patient focus groups and one consisting of 27 questions for the therapist focus groups, with the aim of probing both groups' experience with the usability of the platform. The interview design was based on previous studies (Osma et al. 2022; Titzler et al. 2018), and the draft of both interviews was reviewed by an experienced researcher (J.O.). For its construction, a list of questions representative of each thematic area was designed to select the most relevant ones to be used. The interview with professionals was composed of six main sections, while the interview with users was organized into five sections. Examples of questions from each therapist section and examples from the user interview are presented in Table 2.

**TABLE 2:** Semi‐structured interviews with professionals and users.

Section and Theme	Sample question
***Professionals***	
Phenomenological exploration	
General experience with the platform	In general terms, what did you think of the intervention and the experience using the IUPI platform?
Openness and general background knowledge	Do you usually use apps or technology?
Relationship with other treatments	
Differences with face‐to‐face treatments	What differences might there be between this intervention and face‐to‐face treatments?
Therapist competencies	What competencies should a therapist have to carry out this type of intervention?
Previous experience and knowledge	Did you have prior knowledge of what ICBT is?
Treatment format and structure	
Structure	What do you think of the proposed structure of the different modules?
Interface	What do you think of the interface of the intervention?
Use, access, and understanding	How difficult or easy was it for you to use and access the platform?
Implementation	
Implementation in the local context	What difficulties do you think may arise in the implementation of these interventions in our context?
Public health system	How do you think it could be implemented in the public health system, and what adaptations would need to be made?
Access to treatment	In our country, what barriers to access to treatment do you think may be present in these interventions?
Barriers and facilitators	
Difficulties	What difficulties do you foresee in using the platform with your patients?
Benefits	By using the platform, in your opinion, do you think you could bring any advantage in the treatment of your patients?
Closing and final suggestions	
	In your clinical practice, would you use the intervention as an adjunct to group or individual therapy? Why?
***Users***	
Phenomenological exploration	
General experience with the platform	How was your experience using IUPI?
Openness and prior knowledge	What did you know or know about internet‐based interventions? Would you use them?
Relationship with other treatments	
Differences with face‐to‐face treatments	What differences do you find with face‐to‐face treatments?
Therapist's role	What do you think about the role of the therapist in these interventions?
PU adaptation	What differences do you find in the adaptation of the Unified Protocol for IUPI compared to the group therapy you conducted?
Implementation	
Implementation in the local context	What adaptations do you think they may need to make them work in our context?
Cultural adaptation	Do you think other people would be interested in using the intervention? Why?
Barriers and facilitators	
Difficulties	What disadvantages do you think these interventions have in our context?
Advantages	What advantages do you think the implementation of these interventions has for people in our context?
Closing and final suggestions	Would you recommend IUPI to others in need of treatment? Why?

### 2.7 Data Analysis

Data analysis was carried out with the IBM SPSS statistical package (Version 22.0). For socio‐demographic data, descriptive statistics were calculated. For categorical variables, absolute and percentage frequencies were calculated. For numerical variables, means and standard deviations were calculated.

A thematic analysis of the content of the transcribed interviews was carried out to analyze the information obtained through the focus groups. For this, we followed the fundamentals of Grounded Data Theory (Strauss 1997) and the steps proposed by Maguire and Delahunt (2017), in the framework of the Qualitative Content Analysis methodology (Schreier 2012). The content of the interviews was independently coded by two researchers (MC & FK) from the team in the first instance, identifying emerging themes and sub‐themes based on segments of meaning, and then agreed upon according to their level of saturation. The process was supervised by an experienced team researcher (CG). The qualitative data analysis was carried out using Excel matrices, in which categories, subcategories, and quotes were included independently by each researcher. The Consolidated Criteria for Reporting Qualitative Research (COREQ; see Supporting Information S1: Appendix 3) criteria were followed for reporting the results and analysis.

According to Cohen's Kappa index, independent coding by the two researchers resulted in substantial agreement for users (κ = 0.668) and almost perfect agreement for professionals (κ = 0.808). Thematic saturation was considered reached when no new themes emerged in the groups analyzed. The high level of agreement between independent researchers, along with the process of discussion and comparison until consensus was achieved, ensures that the identified themes and subthemes coherently and comprehensively reflect the data collected.

## 3 Results

### 3.1 Usability Assessment

According to the classification proposed by Bangor et al. (2009), both users (*M* = 73.75, SD = 12.60) and professionals (*M* = 71.37, SD = 19.61) rated the usability of the intervention as good. Table 3 shows the means and standard deviations for each of the items.

**TABLE 3:** SUS means and standard deviations.

Items SUS	Users	Professionals
	Mean (*ED*)	Mean (*ED*)
I would like to use this tool frequently	2.90 (1.37)	2.95 (0.89)
I consider this tool to be unnecessarily complex	2.40 (1.17)	3.10 (0.97)
I find the tool easy to use	3.50 (0.71)	2.90 (0.97)
I consider the support of expert staff necessary to be able to use this tool	2.70 (1.16)	2.40 (1.57)
I consider the functions of the tool to be well‐integrated	2.80 (0.92)	2.75 (0.91)
I consider the tool to have many contradictions	2.90 (0.57)	3.15 (0.86)
I imagine that most people would learn to use this tool quickly	3.00 (0.94)	2.60 (0.10)
I find the use of this tool tedious	2.90 (1.29)	2.85 (1.09)
I felt very confident using the tool	3.10 (0.74)	2.90 (0.79)
I needed to know quite a few things before I could start using this tool	3.30 (0.95)	2.95 (0.89)
SUS total score	73.75 (12.60)	71.38 (19.61)

*Note:* SUS: 72.56; ED: 16.10.

In Table 4, absolute frequencies and percentages are presented on the level of liking and clarity regarding esthetics and design, as well as the level of understanding and information offered on the platform.

**TABLE 4:** Acceptability and satisfaction of the IUPI platform.

Option selected	Users (n = 10)		Professionals (n = 20)	
	*N*	*%*	*N*	*%*
How user‐friendly (in terms of esthetics) did you find the contents of the platform (videos, images, and animations)?				
2	0	0	2	10
3	2	20	7	35
4	2	20	5	25
5	6	60	6	30
How user‐friendly did you find the language used in the videos, images, and animations?				
2	0	0	1	5
3	0	0	4	20
4	2	20	4	20
5	8	80	11	55
How clear did you find the information provided in the videos, images, and animations?				
2	0	0	1	5
3	0	0	1	5
4	3	30	9	45
5	7	70	9	45
Would you use IUPI as an adjunct in therapy/Would you use IUPI as an adjunct in your treatments?				
No	1	10	2	10
Sí	9	90	18	90
Would you recommend others to use IUPI/Would you recommend your patients to use IUPI?				
No	0	0	2	10
Sí	10	100	18	90

*Note:* 1 = unfriendly; 5 = very friendly/1 = unclear; 5 = very clear.

### 3.2 Thematic Analysis

**FIGURE 1:**
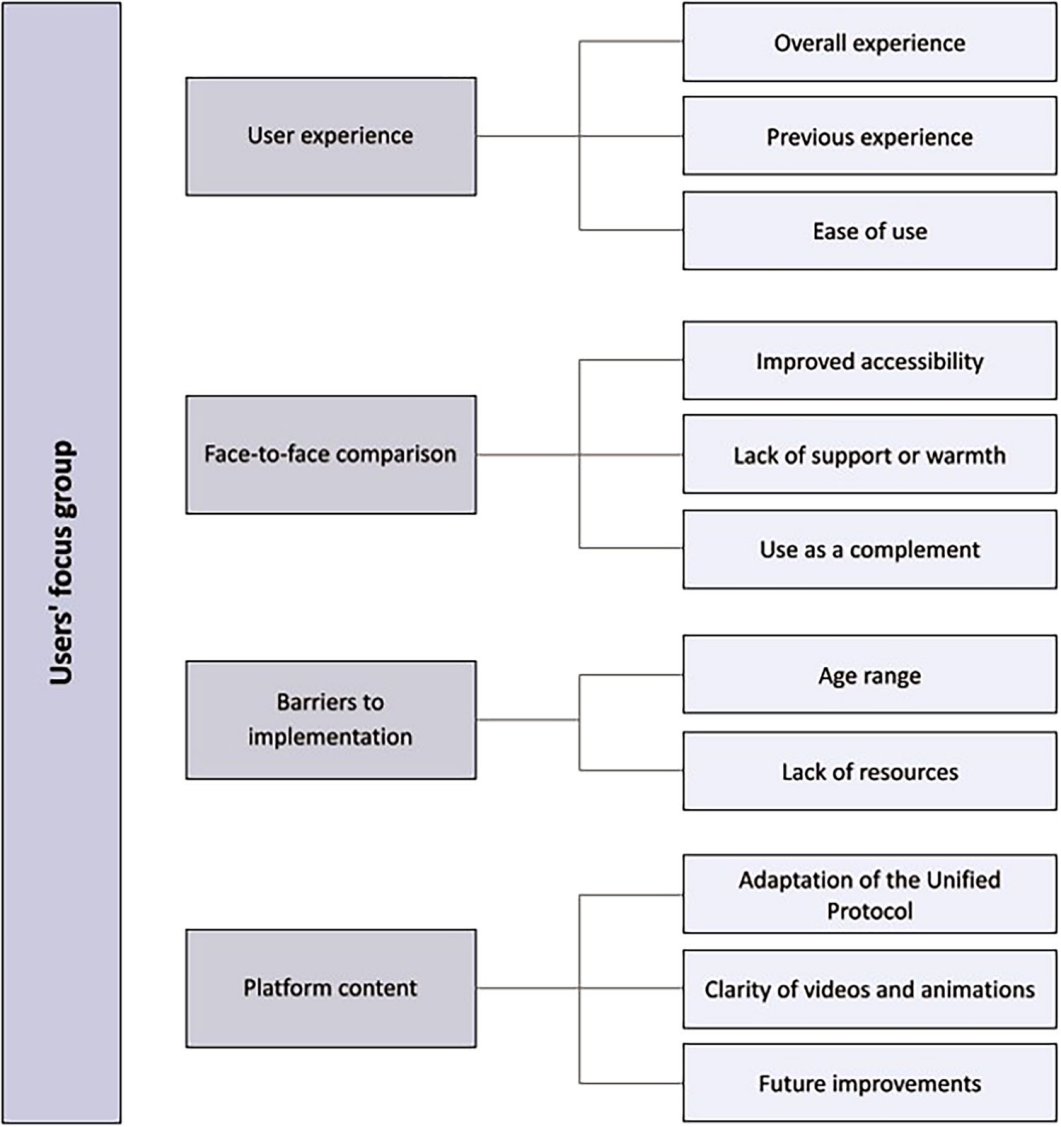
Conformation of user themes and sub‐themes.

**FIGURE 2:**
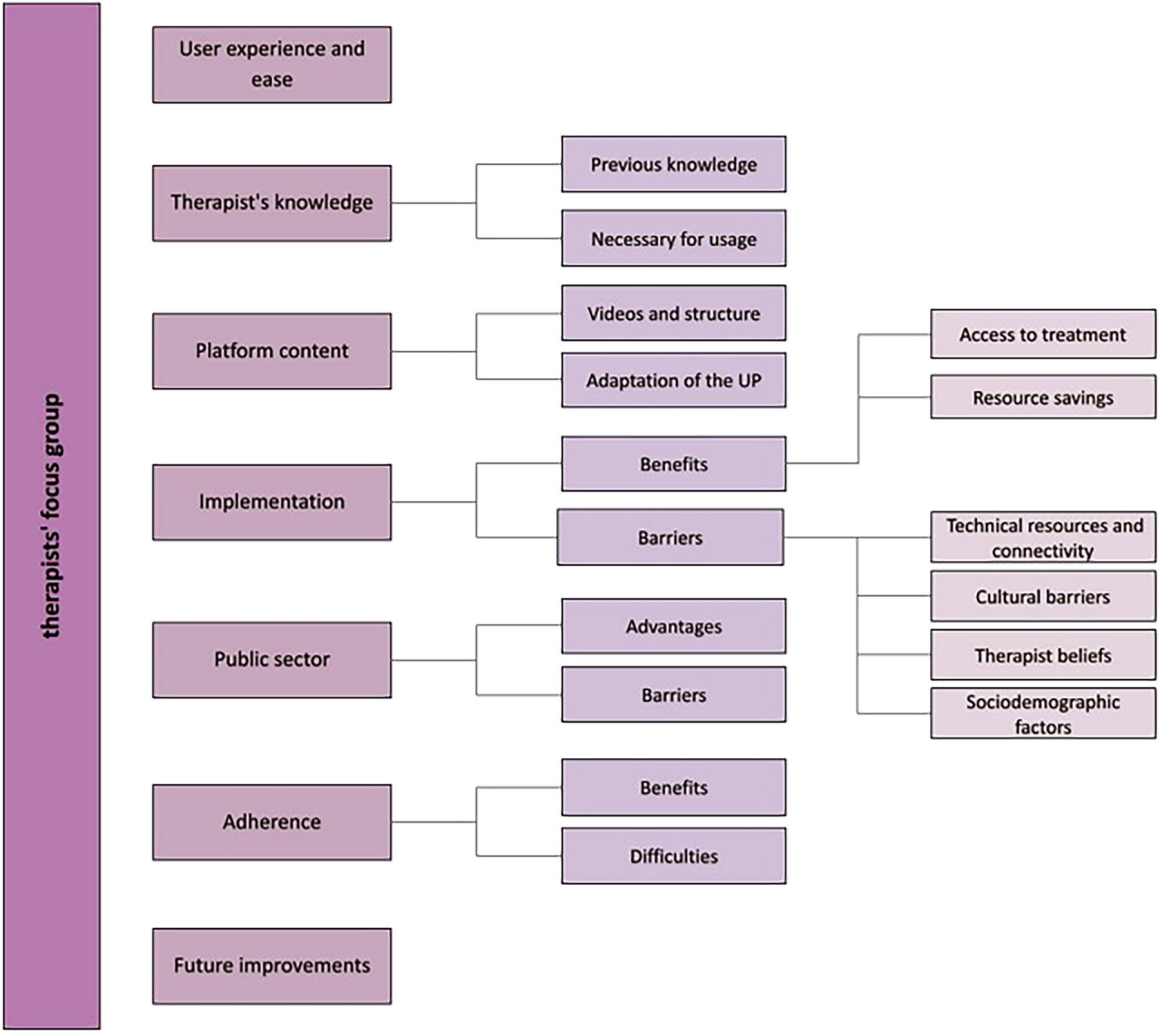
Conformation of professionals themes and sub‐themes.

Results of the analysis of the information collected from the focus group of users and professionals can be seeing in Supporting Information S1: Appendix S1. For the user group, four categories and 11 sub‐categories have been identified, containing 162 code units. For the practitioner focus groups, seven categories and 16 sub‐categories have emerged, and a total of 155 code units. For each of the categories and sub‐categories in each group, a brief description of the content is presented below. In parentheses following each category title, we specify the number of participants who mentioned it. A map of the categories and subcategories of users is presented in Figure 1 and of practitioners in Figure 2 for better understanding.

#### 3.2.1 Thematic Analysis of User Focus Groups

##### 3.2.1.1 User Experience

Within the user experience category, three subcategories have emerged:

###### 3.2.1.1.1 Overall Experience (5/6)

They liked the overall experience of using the IUPI platform, as it was satisfactory and that it was useful. “I thought the application was really good, to be honest. It helped me. I liked all the examples” *(Focus group, participant 3)*, “I felt it was quite complete, having everything there, the scope and also the information that they gave you. It's interesting. It's kind of like you learn something new. So I liked it a lot” *(Focus group, participant 2)*. Emerging in 17.9% of coded units (*n* = 29).

###### 3.2.1.1.2 Previous Experience (6/6)

Users mentioned not being familiar with this type of application, but reported having used or currently using technology for health‐related purposes (e.g., apps for meditation).“I have a meditation application that my psychologist gave me and I don't know if it counts, but it is the only thing” *(Focus group, participant 1)*, “This is the first time I've seen something like this” *(Focus group, participant 2)*. Appearing in 5.6% of coded units (*n* = 9).

###### 3.2.1.1.3 Ease of Use (5/6)

They mentioned that the use of the platform has been intuitive and easy to use, so they have had no problems logging in and using it. “It's very easy, very intuitive to use” *(Focus group, participant 2)*, “I found it quite easy. I mean, no, I didn't have any complications or anything like that” *(Focus group, participant 4)*. Emerging in 9.3% of coded units (*n* = 15).

##### 3.2.1.2 Face‐to‐Face Comparison

In this category, where advantages and disadvantages have emerged about face‐to‐face therapy, three subcategories have been identified:

###### 3.2.1.2.1 Improved Accessibility (6/6)

Users mention that they believe IUPI can improve the accessibility of psychotherapeutic treatments by allowing access to people living in the interior of the country or in remote locations, where it is extremely difficult to access evidence‐based therapies due to a lack of trained therapists. “It's not easy to find a psychologist and the few that there are, perhaps because of the timetable, it's difficult, or from the interior [the countryside] is also good, it's advantageous” (Focus group, participant 3), “Psychologists are also a bit saturated. Post‐pandemic and everything that is happening in the country's situation. I think that having something more at hand or quicker than getting an appointment for a psychologist” (Focus group, participant 2). Emerging in 18.52% of coded units (*n* = 30).

###### 3.2.1.2.2 Lack of Support or Warmth (3/6)

Users mentioned as a disadvantage, compared to face‐to‐face treatments, they perceive that these interventions lack containment, emotional bonding, and exchange with the therapist, which is not generated by using the platform. Some textual examples: “For me, the only disadvantage I see is the coldness of the treatment, let's say, that's why the containment seems important to me. Nothing else, not the rest” *(Focus group, participant 5)*, “yes, maybe sharing with the therapist” (Focus group, participant 6). “Perhaps as a complementary work, perhaps in addition to the face‐to‐face therapy” *(Focus group, participant 5)*, “Yes, I think so, I think so… I recommend it, I would recommend it to these people who don't have…. And I would say that it is also a tool for self‐knowledge” *(Focus group, participant 1)*. Emerging in 6.79% of coded units (*n* = 11).

###### 3.2.1.2.3 Use as a Complement (6/6)

While most participants say they will use IUPI, some of the users comment that they would use and recommend the platform as a complement to other therapies they already do face‐to‐face or online, but where they have direct contact with a therapist. Some textual examples: “Perhaps it is also something more generational. The young people who are today with all the technology, it is perhaps easier to adapt” *(Focus group, participant 6)*, “If it is a person who is very technological or maybe it is a little bit more difficult” *(Focus group, participant 4*). Emerging in 6.17% of coded units (*n* = 11).

##### 3.2.1.3 Barriers to Implementation

Regarding barriers to the implementation of such interventions in Argentina, two subcategories have emerged:

###### 3.2.1.3.1 Age Range (4/6)

One of the difficulties that users perceive in the implementation in our population could be the age range for which it is intended, since they say that young people are more likely to be predisposed to use it because they are the ones who use technology the most. “Perhaps it is also something more generational. The young people who are today with all the technology, it is perhaps easier to adapt” *(Focus group, participant 6)*, “If it is a person who is very technological or maybe it is a little bit more difficult” *(Focus group, participant 4)*. Emerging in 6.79% of coded units (*n* = 11).

###### 3.2.1.3.2 Lack of Resources (4/6)

Another difficulty that emerged according to the participants when implementing this type of tool in our context is the lack of available resources, for example, lack of internet connectivity (or slow/interrupted connectivity), or the lack of technological resources in families, for example, having only one computer. “The issue of not having connectivity” *(Focus group, participant 6)*, “Maybe on a shared computer, for example, in a family home” *(Focus group, participant 4)*. Emerging in 4.32% of coded units (*n* = 7).

##### 3.2.1.4 Platform Content

Regarding the platform's content and potential future improvements, three subcategories have emerged:

###### 3.2.1.4.1 Adaptation of the Unified Protocol (4/6)

Regarding the adaptation made by the UP team to videos, vignettes, and animations, patients who were already familiar with the model reported that the modules are well‐explained and clear, and that the information included is easy to understand. “They were very well explained. Yes, yes, for me it was super step by step, spectacular” *(Focus group, participant 5)*, “The explanation I found it quite easy to understand” *(Focus group, participant 4)*. Emerging in 7.41% of coded units (*n* = 12).

###### 3.2.1.4.2 Clarity of Videos and Animations (4/6)

Participants reported that the videos and animations included were clear, the length appropriate, and the content easy to understand. Some examples: “I thought the length of the videos was good” *(Focus group, participant 6)*, “I also really liked the content of the videos, I think it was all good” *(Focus group, participant 1)*. Emerging in 6.17% of coded units (*n* = 10).

###### 3.2.1.4.3 Future Improvements (6/6)

As for future improvements to the platform, users say they would like it to be usable from a mobile phone (as it is not currently adapted), and they would also like notifications to be included to motivate them to do the exercises. “It's good because you could have notifications. As if to say, well, sit down and do this” *(Focus group, participant 4)*, “Maybe you can think from your mobile phone. It's a good idea” *(Focus group, participant 2)*. Emerging in 11.11% of coded units (*n* = 18).

#### 3.2.2 Thematic Analysis of Focus Groups With Therapists

##### 3.2.2.1 User Experience and Ease (10/16)

Overall, the professionals mentioned that the experience using IUPI was good. They also reported that they found the platform practical and intuitive to use. “The truth is that I liked it, I thought the idea was very good” *(Focus group, participant 13)*, “I thought it was very intuitive, very good, that anyone could do it” *(Focus group, participant 7)*. Emerging in 12.26% of coded units (*n* = 19).

##### 3.2.2.2 Therapist's Knowledge

Regarding the prior knowledge of the therapists and the knowledge required for applying IUPI, two subcategories have emerged:

###### 3.2.2.2.1 Previous Knowledge (7/16)

The professionals mentioned that, at the moment, they have not had any approach to this type of intervention, beyond knowing about them at a theoretical level through reading a book/article or a presentation of the team at a congress. “When I went to the congress, they were mentioned, but I hadn't seen them, I had never come into contact with one and I didn't know what they were like” *(Focus group, participant 4)*, “I might have read about it somewhere, but not specifically like that, no” *(Focus group, participant 7)*. Emerging in 5.16% of coded units (*n* = 8).

###### 3.2.2.2.2 Necessary for Usage (7/16)

In this category, professionals mention that, for the implementation and correct use of this type of intervention by therapists, further training in digital technologies is required. At the same time, they mention that other requirements could be a strong background in behavioral therapy and UP. “We also need to have more knowledge and training in technology” *(Focus group, participant 7)*, “To be trained in the protocol, I think it is important” *(Focus group, participant 1*). Emerging in 5.16% of coded units (*n* = 8).

##### 3.2.2.3 Platform Content

In the category of platform content, two subcategories have emerged:

###### 3.2.2.3.1 Videos and Structure (9/16)

As for the content of the videos, the professionals report that they are clear in terms of the explanations of the concepts, as well as the animations. On the other hand, the structure of the platform has been tidy and clear. “All those animations were very good” *(Focus group, participant 4)*, “Very easy access to each module, and everything was very well organized” *(Focus group, participant 3*). Emerging in 12.26% del codigo (*n* = 19).

###### 3.2.2.3.2 Adaptation of the UP (6/16)

Regarding the adaptation of the UP to the web platform, the professionals who know and use it say that it was faithful to the model and completed the adaptation. On the other hand, they also mention that in some cases, being a structured protocol, it can be inflexible. “Exactly what I imagined, I thought it was very complete. I thought it was going to be more summarized, let's say, that it was going to have some aspects, and it seemed to me that it was quite respectful of what appears in the modules” *(Focus group, participant 9)*. “It's very structured, I'm just going to say that, maybe some people find it very, very structured, but it's a good tool” *(Focus group, participant 3)*. Emerging in 4.52% of coded units (*n* = 7).

##### 3.2.2.4 Implementation

Regarding the implementation of such interventions in our context, two main subcategories and six infra‐categories have emerged:

###### 3.2.2.4.1 Benefits

Regarding the potential benefits, two key points are mentioned:

Access to treatment (9/16). On the one hand, professionals mention that they facilitate greater access to psychotherapeutic treatments, as access to evidence‐based practices is often complex due to lack of financial resources. “It will be much more accessible to access psychotherapy in evidence for people who, perhaps, we all know how difficult it is to access psychotherapeutic treatment” *(Focus group, participant 10)*. “That more people at the same time can have access to treatment, which we know is very difficult to access financially as well” *(Focus group, participant 16)*. Emerging in 9.68% of coded units (*n* = 15). Resource savings (5/16). Another benefit mentioned by professionals is the saving of resources. Through this type of intervention, it might be possible to reach a larger number of patients in a shorter time, also making access to psychotherapy more accessible. “It covers a larger number of people. I also think I can massify a bit more or reach those places” *(Focus group, participant 1)*. “It's a very good tool for us so that we don't have to spend an hour with a patient, and we can do more work” (Focus group, participant 11). Emerging in 4.52% of coded units (*n* = 7).

###### 3.2.2.4.2 Barriers

On the other hand, professionals mention a series of barriers:

Technical resources and connectivity (6/16). The lack of connectivity and technical resources in rural areas, or especially in the interior of the country, was mentioned by professionals as one of the main barriers to implementation, given that not all the population has access to computers or an internet connection. “Connectivity, I feel that, for example, I have patients in Santiago del Estero who sometimes do not have the best accessibility too, because I am from Santiago del Estero, I know the place, and there are places where the signals are sometimes very complex…. I think that connectivity is one of the biggest barriers” *(Focus group, participant 2)*, “We don't have the tools to see connectivity at home, I am from Mendoza, I don't know if they are all from Buenos Aires, it is very scarce in the population” *(Focus group, participant 6)*. Emerging in 7.74% of coded units (*n* = 12).

####### 3.2.2.4.2.1 Cultural barriers (8/16)

On the other hand, cultural aspects related to face‐to‐face contact and a lack of human connection are mentioned as a barrier. They also refer to the strong influence of the psychoanalytic tradition in psychotherapy that characterizes the country. “Culturally, we are more about contact and face‐to‐face contact. I think that this also affects our culture a bit” *(Focus group, participant 6)*. “The patients, we are in a culture very marked by this, of the psychoanalytic and the importance of talking” *(Focus group, participant 11)*. Emerging in 10.32% of coded units (*n* = 16).

####### 3.2.2.4.2.2 Therapist beliefs (11/16)

Practitioners also refer as a barrier to the beliefs or preconceptions that are often held about this type of intervention, for example, that the craft of psychotherapy is lost, that it is superficial or that it is lost on a case‐by‐case basis. “In that sense, I feel that perhaps it takes away a bit of the more artisanal part of the therapist modifying the structure of the different protocols for each patient”. *(Focus group, participant 2)*. “You lose the case by case because it's like you're reading what someone else answered, it sounds a bit superficial” *(Focus group, participant 13)*. Emerging in 14.83% of coded units (*n* = 23).

####### 3.2.2.4.2.3 Sociodemographic factors (age, education; 6/16)

In terms of socio‐demographic variables, they suggest that both age (where the older the person is, the less inclined they are to use them) and educational level may result in barriers to access in our context. “The issue of also the level of education to understand all the instructions well. Maybe that can also be a barrier” *(Focus group, participant 8)*. “Surely there are still older patients where they may not get so confused with the use of an application” *(Focus group, participant 2)*. Emerging in 5.8% of coded units (*n* = 9).

###### 3.2.2.4.3 Public Sector

Within the topic of the public sector, two subcategories have emerged:

####### 3.2.2.4.3.1 Advantages (4/16)

In this category, participants working in the public sector commented that the advantages of implementing an intervention such as IUPI could be useful due to the huge waiting lists that hospitals currently have, as patients do not receive any treatment in that time. “I think it would be very useful in the public sector because of the amount of demand, which is impressive in a public hospital, for example, it is impressive” *(Focus group, participant 3)*. “I'm speaking from the public hospital, but I think it could be implemented, and I think it happens a lot, that a lot of people arrive without treatment and they can't get it anywhere, and the whole system is saturated” *(Focus group, participant 16)*. Emerging in 3.87% of coded units (*n* = 6).

####### 3.2.2.4.3.2 Barriers (4/16)

In terms of barriers to implementation in the public context, practitioners report that there may be resistance from hospital staff, especially senior professionals. “Well, I work in a public hospital, so there is also something about technology that is very difficult for us to implement and that would be great because it would help a lot, but we are a bit alien to it” *(Focus group, participant 16)*. “I also think that public hospital professionals, perhaps not so much as us residents who are generally younger and more up to date, have certain defenses in this regard” *(Focus group, participant 16)*. Emerging in 2.58% of coded units (*n* = 4).

###### 3.2.2.4.4 Adherence

Within this category, two subcategories have emerged:

####### 3.2.2.4.4.1 Beneficios (6/16)

On the one hand, practitioners report benefits that these interventions can bring in terms of adherence to such interventions and also in combination with face‐to‐face treatments, for example, by allowing greater utilization and availability of records. “The records are great, it makes it much easier to fill in the forms, with boxes so that the person can do it directly from their mobile phone or computer. In that sense, it's a great thing” *(Focus group, participant 2)*. “Above all, for patients in fortnightly mode, I think it's a good way to help them keep track” *(Focus group, participant 9)*. Emerging in 4.52% of coded units (*n* = 7).

####### 3.2.2.4.4.2 Difficulties (5/16)

On the other hand, professionals mention possible challenges in terms of treatment adherence. They argue that this type of intervention lacks the commitment of a scheduled appointment with the therapist, which may reduce motivation to undertake the treatment. “It may be more of a procrastination issue, or it may be an encouragement of procrastination” *(Focus group, participant 15)*. “Maybe motivation can go down and you don't want to continue. For example, I think it could be a disadvantage because it is a bit long, so motivation could drop” *(Focus group, participant 3*). Emerging in 3.22% of coded units (*n* = 5).

###### 3.2.2.4.5 Future Improvements (9/16)

In this category, several proposals for the future improvement of the application are presented. On the one hand, the professionals mention that it would be necessary to adapt it so that it can be used on mobile devices; and on the other hand, they also mention that improvements could be made in terms of esthetics, such as more colors or more eye‐catching buttons. “It seems to me that some aspects of attractiveness could be improved” *(Focus group, participant 7)*. “I feel that it would be somewhat counterproductive for an application to be more complex from the mobile phone than from the computer, as it is very likely that some patients, particularly the majority, tend to connect from the mobile phone” *(Focus group, participant 2)*. Emerging in 9.03% of coded units (*n* = 14).

### 3.3 Discussion

The study aims to assess the usability and acceptability of the IUPI platform among users and mental health professionals in Argentina. To the best of our knowledge, this is the first study to evaluate an ICBT created entirely in our country. The results demonstrate that IUPI has good acceptability within the Argentinean population. These findings are encouraging for the development and implementation of such interventions, as perceived usability and usefulness are essential for their adoption, dissemination, and scalability (Harst et al. 2019).

Regarding the results obtained from the SUS scale, both groups indicate good acceptability of the intervention, with users (*M* = 73.75, SD = 12.60) and professionals (*M* = 71.37, SD = 19.61), showing similar scores, slightly higher in users. Both scores meet the minimum desirable usability threshold of > 70 (Bangor et al. 2008) and exceed the average of 68 suggested by Sauro and Lewis (2016). The results are even slightly superior to other internet‐based interventions studied in LMICs (Kheirkhah et al. 2023; Rahmadiana et al. 2021), highlighting the population's openness to this type of intervention. In line with this, thematic analysis shows that most users and professionals express a willingness to use it.

Regarding users, the highest‐rated item was “*I consider the tool easy to use*” while the lowest was “*I consider this tool unnecessarily complex*” This aligns with the qualitative analysis, where participants highlighted the platform's ease of use even though none were familiar with such interventions before. It also aligns with 100% of users recommending the intervention and 90% using it again, indicating strong willingness to adopt it. Additionally, users found the app friendly and the information clear, suggesting high usability and acceptability. These findings are promising for implementation in our country, as low usability is a reported barrier to ICBT adoption (Folker et al. 2018), and a positive relationship between usability and perceived utility is linked to subsequent effectiveness (Mira et al. 2019).

From the professionals' perspective, the thematic analysis indicates that their overall experience with the application was positive, reporting that it was easy to use. This matches the overall SUS score but contrasts with higher‐rated items such as “*I believe the tool has many contradictions*” and “*I believe this tool is unnecessarily complex.*” These results appear contradictory when compared to the user responses and may reflect an overestimation of the platform's complexity by therapists, possibly due to their lack of previous experience using such interventions in clinical practice (Bunge et al. 2009). We also consider that this may be partially explained by an initial resistance to digital interventions, given Argentina's cultural preference for face‐to‐face interactions, an aspect further discussed in the following sections. The thematic analysis and previous literature (Titzler et al. 2018) highlight the need for specific therapist training to ensure proper implementation. On the other hand, the item with the lowest mean score was *“I think expert support is necessary to use this tool”* which is consistent with the findings from the thematic analysis, where users reported being able to use the platform without difficulty.

Regarding the adaptation of the UP, users and professionals with prior knowledge of the intervention reported that it was faithful, and the explanations were easy to understand. Professionals reported that it might be too structured, in line with previous literature (Titzler et al. 2018), noting that these interventions are not very customizable, limiting their dissemination.

The professionals showed a high willingness to implement IUPI, with 90% expressing that they would recommend it to their patients and use it as a complement to their treatments. This could be attributed to their previous experience with the intervention, providing them with a certain understanding of its usage. However, the thematic analysis reveals negative beliefs, such as the perception that these interventions are superficial, as a barrier to implementation, consistent with previous research about negative attitudes from therapists as a barrier (Folker et al. 2018), and therapists' willingness to use teletherapy tools depends on their perceived usefulness (Monthuy‐Blanc et al. 2013).

Users and professionals agree that ICBT enhances accessibility to treatments and emphasizes the potential for time and human resource savings, enabling the treatment of more patients in less time. This is particularly relevant in Argentina, where the ongoing economic crisis limits access to psychotherapy due to financial constraints and a shortage of professionals in the public healthcare system (Bureau et al. 2021; Etchevers et al. 2021; Klinar et al. 2019; Titzler et al. 2018).

Some users and professionals mentioned that these interventions felt cold or lacked emotional support, aligning with a previous study (Appeceix et al. 2024). This may be explained by the sociocultural characteristics of the population (Korman and Roche 2019; Korman et al. 2022; Korman and Sarudiansky 2023), given the strong influence of psychoanalysis. In addition, it has been studied that adherence is lower in unguided interventions (Zhang et al. 2024). In this regard, the thematic analysis indicates that most users would use it as a complement to another therapy (i.e., maintaining some in‐person contact), known as a blended format (Mathiasen et al. 2016; Osma et al. 2021). This format emerges as a possible alternative to the high dropout rates observed in ICBT (Bendelin et al. 2011; Esser et al. 2023). This finding is consistent with what professionals mentioned, noting that adherence could be a problem due to the lack of motivation caused by the therapist's absence. Previous qualitative studies with patients reported the lack of contact with the therapist as the main reason for dropout (Karyotaki et al. 2022; Titzler et al. 2018), and in general, therapist‐guided interventions showed better effectiveness (Zhang et al. 2024).

Age emerged in thematic analysis as another access barrier due to the generational gap, as both professionals and users indicated that younger individuals might be more willing to use these interventions. The evidence on this issue is controversial. Castro et al. (2017) found that older age predicted greater adherence, while Christensen et al. (2009) found that younger age was associated with higher adherence. Future studies are expected to provide more context‐specific evidence.

Other perceived barriers include the lack of resources, such as difficulty with internet connectivity, which has been reported in previous studies in similar countries (Rahmadiana et al. 2021) and is common in remote areas of Argentina. According to the latest available data, approximately 55% of Argentines are active internet users (World Health Organization, WHO 2016). They also mention the availability of computers, as in rural areas, people may only have a mobile phone or a shared family computer. Additionally, professionals highlighted that educational level could present an access gap, known as the digital gap (García Vargas et al. 2020), a persistent problem in Latin America (Alderete and Formichella 2016).

Professionals in the public sector highlighted the potential of IUPI to reduce existing limitations, especially the long waiting lists in a collapsed system. According to official data, 35% of the population use the public healthcare system (Congress of the Nation 2021), which has a decentralized structure (national, provincial, and municipal funding) and significant challenges in resource redistribution (Novick 2017). These issues result in health spending inequalities and system saturation due to insufficient professionals (Palacios et al. 2020). Professionals mention obstacles to implementation, such as unfamiliarity with technology among many professionals, consistent with previous studies (Bunge et al. 2009), and potential resistance to change. We hypothesize that this resistance may be linked to a lack of funding in the sector and limited technological resource implementation.

Regarding future improvements, it is essential to incorporate user feedback in the design of ICBT, as this enhances usability and subsequent implementation (Martínez‐García et al. 2024; Skivington et al. 2021). Users and professionals emphasize the need for the intervention to be mobile‐compatible, a good way to improve accessibility, since it is estimated that there is more than one mobile phone internet subscription per person in Argentina (World Health Organization, WHO 2016). Another proposed future enhancement is the possibility of using the platform offline by downloading activities. The ability to operate on mobile devices and offline is expected to help bridge the technology access gap (García Vargas et al. 2020). Based on the results obtained, in LMIC, key factors for the adoption of such interventions may include prioritizing mobile app development, enabling online and downloadable functionality, and simplifying content. Another suggested improvement raised by users involves implementing platform‐generated notifications as reminders. Currently, the team is working on adapting IUPI for mobile devices and integrating notification features for the next update. A further enhancement proposed based on this study's findings is conducting a *blended* version pilot test of the platform, considering that one of the identified barriers relates to perceived lack of interpersonal warmth, a challenge that may also apply to countries with similar cultural characteristics.

To conclude, this study presents some limitations that should be mentioned. On the one hand, the sample users who agreed to participate in the research are predominantly (90%) university‐educated, which is not very representative, given that the population over 25 years old with a completed university education is around 20% (Jorrat et al. 2024). This makes it difficult to generalize the results obtained, particularly to populations with lower educational levels and presumably fewer economic resources. In turn, due to the sampling method used (snowball sampling), the responses may be favorably biased, although attempts were made to reduce bias through sample diversity. In future studies, sample stratification is expected. On the other hand, the SUS has not yet been adapted to the local context, but rather the Spanish version adapted to Spanish has been used (Sevilla‐Gonzalez et al. 2020), which could reduce the reliability of the instrument used. Future research hopes to have the scale validated in the local population to ensure its linguistic and cultural validity. An additional limitation of this study is that during this initial usability test, we were unable to collect data on the exact number of activities completed by each participant on the platform. In future trials, we plan to capture this information to ensure greater data fidelity. Finally, it should be noted that the discourse saturation method (Guest et al. 2020) could not be employed in this initial test. Future studies hope to incorporate more robust qualitative research methods.

Although further research is needed, IUPI appears to be acceptable and feasible. This study provides an initial contribution to the implementation of these interventions, and future studies will continue with a randomized controlled trial to assess the efficacy of the intervention in reducing EDs' symptoms. This will improve access to treatments in low‐resource settings, providing a step toward making these interventions publicly available in Argentina.

## Author Contributions

J. O. and M. C. contributed to the study's conception and design. M. C., F. K., and C. C. conducted material preparation, data collection, and analysis. M. C. wrote the first draft of the manuscript, while F. K. prepared figures and tables. J. O. and C. G. handled writing reviews and editing. Funding was obtained by C. G., M. E. and J. O. All authors reviewed earlier versions of the manuscript and provided feedback. The final manuscript was read and approved by all authors.

## Ethics Statement

This study has been approved by the Committee for Responsible Conduct in Research of the Faculty of Psychology at the University of Buenos Aires.

## Consent

Informed consent was obtained from all participants included in the study. No person's data (individual details, images or videos) are reported in this manuscript.

## Supporting information

Appendix 1.

Appendix 2.

Appendix 3.

Appendix 4.

## Data Availability

The data supporting this study's findings are not openly available due to sensitivity reasons but are available from the corresponding author upon reasonable request.
