# Supplementary material for: Usability and Acceptance by Therapists and Users of an Internet‐Based Intervention Based on the Unified Protocol in Argentina
Source: J Clin Psychol. 2025 Aug 20;81(12):1294–309. doi: 10.1002/jclp.70037 (PMC12598383; doi:10.1002/jclp.70037)
Supplement: Supplementary file 1 — Appendix 1. [file JCLP-81-1294-s002.docx]

**Appendix 1**

*Good Reporting of a Mixed Methods Study (GRAMMS)*

| **Guidelines** | **Section: page** |
| --- | --- |
| Rationale for the use of a mixed methods approach to research | Method (Design): p. 4 |
| Description of the design in terms of purpose, priority and sequence of methods | Method: p. 4 |
| Description of each method in terms of sampling, data collection and analysis | Method:  Participants: pp. 5-6  Instruments: p. 4-5  Data analysis: p. 7 |
| Description of where the integration has taken place, how it has taken place and who has been involved in it | Method (Design): p. 4 |
| Description of limitations of one method associated with the presence of another. | Method (Design): p. 4 |
| Description of knowledge gained from a mixed-methods approach | Discussion: p. 11-12 |

*Note:* O'Cathain, A., Murphy, E., & Nicholl, J. (2008). The quality of mixed methods studies in health services research*. Journal Health Services Research & Policy.* 13(2):92-98.
