# Supplementary material for: Usability and Acceptance by Therapists and Users of an Internet‐Based Intervention Based on the Unified Protocol in Argentina
Source: J Clin Psychol. 2025 Aug 20;81(12):1294–309. doi: 10.1002/jclp.70037 (PMC12598383; doi:10.1002/jclp.70037)
Supplement: Supplementary file 2 — Appendix 2. [file JCLP-81-1294-s004.docx]

**Appendix 2**

*Contents of the beta version of the IUPI platform*

| Session number | | PU module | Examples of content and exercises |
| --- | --- | --- | --- |
|  | *Session 1* | Module 0: Introduction to the program, therapists, and the functional model of emotional disorders. | Animation: Carla (fictitious character) tells what happens to her in the framework of the functional model. Exercise: Complete your functional model. |
|  | *Session 2* | Module 1: Setting goals and maintaining motivation. | Video: Therapeutic goals and their characteristics are introduced. The therapist explains the fluctuation of motivation and introduces the decision balance.  Exercise: Completing goals and steps to achieve them and completing the costs of changing and staying the same. |
|  | *Session 3* | Module 2: Understanding Emotions | Video: introduction to the three-component model and examples of the interaction between them. The ARCO register and the functional analysis of emotions are introduced.  Exercise: decompose an intense emotion with an example from last week and complete the ARCO register. |
|  | *Session 4* | Module 3: Mindfulness | Audio: guided mindfulness exercise. Video: The therapist explains anchoring in the present and emotional induction exercises with videos presented.  Exercise: Complete the emotional mindfulness log (what did you notice?). Practice observing emotions and recording emotional responses. |
|  | *Session 5* | Module 4: Cognitive Flexibility | Video: The therapist introduces ambiguous image exercise. Thought traps and questioning of automatic thoughts are introduced. The downward arrow is explained. Exercise: Perform three alternative interpretations of the ambiguous image. Record thoughts and question them. |
|  | *Session 6* | Module 5: Emotional Behaviours | Animation: Several examples of different types of avoidance (overt, subtle, cognitive) and their short- and long-term consequences are introduced. The concept of alternative action and its consequences in the short and long term are introduced.  Exercise: identifying one's emotional behaviors and alternative actions. |
|  | *Session 7* | Module 6: Coping with physical sensations | Video: The therapist introduces the role of physical sensations and the practice of interoceptive exposure exercises.  Exercise: completing and identifying the physical sensations experienced. |
|  | *Session 8* | Module 7: Emotional exposure | Video: The therapist introduces the construction of exposure hierarchy. The aim of exposure and why it is important for the treatment is explained. Pre-exposure and post-exposure recording are exemplified.  Exercise: Complete your exposure hierarchy and start exposing yourself during the week. |
|  | *Session 9* | Module 8: Recognising achievements, preventing relapses and closing the program. | Video: The therapist recapitulates the tools learned during the program. The concept of fall and relapse is explained. The importance of continuing the practice is described.  Exercise: Identify achievements and tools learned. Identify how skills will continue to be practiced. |
