# Supplementary material for: Usability and Acceptance by Therapists and Users of an Internet‐Based Intervention Based on the Unified Protocol in Argentina
Source: J Clin Psychol. 2025 Aug 20;81(12):1294–309. doi: 10.1002/jclp.70037 (PMC12598383; doi:10.1002/jclp.70037)
Supplement: Supplementary file 3 — Appendix 3. [file JCLP-81-1294-s001.docx]

| **Appendix 3**  *Consolidated criteria for reporting qualitative studies (COREQ): 32-item checklist* | | |
| --- | --- | --- |
| **Item** | **Guide questions/description** | **Reported** |
| **Domain 1: Research team and reﬂexivity** | | |
| *Personal Characteristics* | | |
| 1. Interviewer/ facilitator | Which author/s conducted the interview or focus group? | MC  FK |
| 2. Credentials | What were the researcher’s credentials? E.g. PhD, MD | PhD Student (MC)  Undergraduate student (FK) |
| 3. Occupation | What was their occupation at the time of the study? | PhD Student. UBACYT Research Trainee (MC).  UBACYT stimulus grant holder (FK). |
| 4. Gender | Was the researcher male or female? | Female |
| 5. Experience and training | What experience or training did the researcher have? | MC has 5 years of experience in quality-quantitative research and as a cognitive therapist.  FK has one year of experience assisting the research team.  Both have completed a webinar on grounded theory in the current year. |
| *Relationship with participants* | | |
| 6. Relationship established | Was a relationship established prior to study commencement? | MC coordinated therapy groups in which some of the participants of the user group (users with previous PU experience) participated. |
| 7. Participant knowledge of the interviewer | What did the participants know about the researcher? e.g. personal goals, reasons for doing the research | The participants knew the institutional affiliations of the researchers.  Personal goals: none.  The reasons for doing the interviews were known and stated at the beginning of the interviews. |
| 8. Interviewer characteristics | What characteristics were reported about the interviewer/facilitator? e.g. Bias, assumptions, reasons, and interests in the research topic | Participants were told that the interviewer is interested in the feasibility and future implementation of the platform and the interview aimed to get insights into relevant factors to use for later implementation projects both in private practice and public healthcare |
| **Domain 2: study design** |  |  |
| *Theoretical framework* |  |  |
| 9. Methodological orientation and Theory | What methodological orientation was stated to underpin the study? e.g. grounded theory, discourse analysis, ethnography, phenomenology, content analysis | Grounded theory  Deductive Content Analysis |
| *Participant selection* |  |  |
| 10. Sampling | How were participants selected? e.g. purposive, convenience, consecutive, snowball | Purposive, snowball |
| 11. Method of approach | How were participants approached? e.g. face-to-face, telephone, mail, email | Email, social networks, and dissemination among colleagues. Participants of therapeutic groups were also contacted. |
| 12. Sample size | How many participants were in the study? | 30 participants |
| 13. Non-participation | How many people refused to participate or dropped out? Reasons? | 4 participants reported unavailability to participate in the focus group; 21 dropped out unexplained. |
| *Setting* |  |  |
| 14. Setting of data collection | Where was the data collected? e.g. home, clinic, workplace | Internet |
| 15. Presence of non-participants | Was anyone else present besides the participants and researchers? | No |
| 16. Description of sample | What are the important characteristics of the sample? e.g. demographic data, date | Cognitive-behavioral therapists from all over the country. People with self-referred symptoms of anxiety or depression, or who have participated in a therapeutic group of the team. |
| *Data collection* |  |  |
| 17. Interview guide | Were questions, prompts, guides provided by the authors? Was it pilot tested? | Yes. No pilot test done. |
| 18. Repeat interviews | Were repeat interviews carried out? If yes, how many? | No |
| 19. Audio/visual recording | Did the research use audio or visual recording to collect the data? | Audio recording was used |
| 20. Field notes | Were ﬁeld notes made during and/or after the interview or focus group? | Yes, field notes were made during the interview |
| 21. Duration | What was the duration of the interviews or focus group? | M = 59.6 minutes, SD = 15.8, Min = 44 minutes, Max = 83 minutes |
| 23. Transcripts returned | Were transcripts returned to participants for comment and/or correction? | No |
| **Domain 3: analysis and ﬁndings** | | |
| *Data analysis* |  |  |
| 24. Number of data coders | How many data coders coded the data? | 2 |
| 25. Description of the coding tree | Did authors provide a description of the coding tree? | Yes |
| 26. Derivation of themes | Were themes identiﬁed in advance or derived from the data? | Derived from the data |
| 27. Software | What software, if applicable, was used to manage the data? | Microsoft Excel |
| 28. Participant checking | Did participants provide feedback on the ﬁndings? | No |
| *Reporting* |  |  |
| 29. Quotations presented | Were participant quotations presented to illustrate the themes/ﬁndings? Was each quotation identiﬁed (e.g. ID)? | Yes |
| 30. Data and ﬁndings consistent | Was there consistency between the data presented and the ﬁndings? | Yes |
| 31. Clarity of major themes | Were major themes clearly presented in the ﬁndings? | Yes |
| 32. Clarity of minor themes | Is there a description of diverse cases or discussion of minor themes? | Yes |
