# Supplementary material for: Usability and Acceptance by Therapists and Users of an Internet‐Based Intervention Based on the Unified Protocol in Argentina
Source: J Clin Psychol. 2025 Aug 20;81(12):1294–309. doi: 10.1002/jclp.70037 (PMC12598383; doi:10.1002/jclp.70037)
Supplement: Supplementary file 4 — Appendix 4. [file JCLP-81-1294-s003.docx]

**Appendix 4**

*Results of the analysis of the information collected from the focus group of users and professionals.*

|  | **Categories** | **Subcategories** | **Description** | **Example** |
| --- | --- | --- | --- | --- |
| **Users** | User experience | Overall experience | General experience with the platform. | “I thought the application was really good, to be honest. It helped me. I liked all the examples” (Focus group, participant 3), “I felt it was quite complete, having everything there, the scope and also the information that they gave you. It's interesting. It's kind of like you learn something new. So I liked it a lot” (Focus group, participant 2). |
|  |  | Previous experience | Prior knowledge of internet-based interventions. | “I have a meditation application that my psychologist gave me and I don't know if it counts, but it is the only thing” (Focus group, participant 1), “This is the first time I've seen something like this” (Focus group, participant 2). |
|  |  | Ease of use. | Ease of accessing and navigating the platform. They have had no problems when logging in and being able to use it. | “It's very easy, very intuitive to use” (Focus group, participant 2), “I found it quite easy. I mean, no, I didn't have any complications or anything like that” (Focus group, participant 4). |
|  | Face-to-face comparison. | Improved accessibility | Positive perception of greater access to psychotherapy through face-to-face intervention by allowing access to people living in the interior of the country or in remote locations, where it is extremely difficult to access evidence-based therapies due to a lack of trained therapists. | “It's not easy to find a psychologist and the few that there are, perhaps because of the timetable, it's difficult, or from the interior [the countryside] is also good, it's advantageous” (Focus group, participant 3), “Psychologists are also a bit saturated. Post-pandemic and everything that is happening in the country's situation. I think that having something more at hand or quicker, than getting an appointment for a psychologist” (Focus group, participant 2). |
|  |  | Lack of support or warmth | They mention that these interventions lack emotional support or warmth relative to face-to-face. | “For me, the only disadvantage I see is the coldness of the treatment, let's say, that's why the containment seems important to me. Nothing else, not the rest” (Focus group, participant 5), “yes, maybe sharing with the therapist” (Focus group, participant 6). |
|  |  | Use as a complement. | Some of the users commented that they would use and recommend the platform as a complement to other therapies that they already do in person or online, but where they have direct contact with a therapist. | “Perhaps as a complementary work, perhaps in addition to the face-to-face therapy” (Focus group, participant 5), “Yes, I think so, I think so... I recommend it, I would recommend it to these people who don't have.... And I would say that it is also a tool for self-knowledge” (Focus group, participant 1). |
|  | Barriers to implementation | Age range | One of the challenges users perceive in implementing this intervention in our population could be the target age range. | “Perhaps it is also something more generational. The young people who are today with all the technology, it is perhaps easier to adapt” (Focus group, participant 6), “If it is a person who is very technological or maybe it is a little bit more difficult” (Focus group, participant 4). |
|  |  | Lack of resources | They mention the lack of available resources. Examples include insufficient or unreliable Internet connectivity and limited access to technological devices within families. | “The issue of not having connectivity” (Focus group, participant 6), “Maybe on a shared computer, for example, in a family home” (Focus group, participant 4). |
|  | Platform content | Adaptation of the Unified Protocol | They mention that the adaptation of the protocol was faithful to the manual. | “They were very well explained. Yes, yes, for me it was super step by step, spectacular” (Focus group, participant 5), “The explanation I found it quite easy to understand” (Focus group, participant 4). |
|  |  | Clarity of videos and animations | The included videos and animations were clear of appropriate length | “I thought the length of the videos was good” (Focus group, participant 6), “I also really liked the content of the videos, I think it was all good” (Focus group, participant 1). |
|  |  | Future improvements | Suggestions and proposals to improve the platform | “It's good because you could have notifications. As if to say, well, sit down and do this” (Focus group, participant 4), “Maybe you can think from your mobile phone. It's a good idea” (Focus group, participant 2). |
|  | **Categories** | **Subcategories** | **Description/Subcategories** | **Example** |
| **Professionals** | User experience and ease | | The use of the Platform is practical and intuitive in global terms. | “The truth is that I liked it, I thought the idea was very good” (Focus group, participant 13), “I thought it was very intuitive, very good, that anyone could do it” (Focus group, participant 7). |
|  | Therapist's knowledge | Previous knowledge | Previous approaches to this type of intervention. Most of the participants only knew about them at a theoretical level. | “When I went to the congress, they were mentioned, but I hadn't seen them, I had never come into contact with one and I didn't know what they were like” (Focus group, participant 4), “I might have read about it somewhere, but not specifically like that, no” (Focus group, participant 7). |
|  |  | Necessary for usage | They refer to the specific training necessary for the use of the intervention. | “We also need to have more knowledge, and training in technology” (Focus group, participant 7), “To be trained in the protocol, I think it is important” (Focus group, participant 1). |
|  | Platform content | Videos and structure | They note that the platform's structure has been well-organized and clear. | “All those animations were very good” (Focus group, participant 4), “Very easy access to each module, and everything was very well organized” (Focus group, participant 3). |
|  |  | Adaptation of the PU | They report that it is faithful to the model and that the adaptation is complete. Also mention that in some cases, as a structured protocol, it can be inflexible. | “Exactly what I imagined, I thought it was very complete. I thought it was going to be more summarized, let's say, that it was going to have some aspects, and it seemed to me that it was quite respectful of what appears in the modules” (Focus group, participant 9). “It's very structured, I'm just going to say that, maybe some people find it very, very structured, but it's a good tool” (Focus group, participant 3). |
|  | Implementation | *Benefits*  Positive aspects for use and implementation of the intervention | *Access to treatment:*  Greater access to psychotherapeutic treatments, as accessing evidence-based practices is often challenging due to a lack of financial resources. | “It will be much more accessible to access psychotherapy in evidence for people who perhaps we all know how difficult it is to access psychotherapeutic treatment” (Focus group, participant 10). “That more people at the same time can have access to treatment, which we know is very difficult to access financially as well” (Focus group, participant 16). |
|  |  |  | *Resource savings:*  Through this type of intervention, it could be possible to reach a larger number of patients in less time, also making access to psychotherapy more accessible. | “It covers a larger number of people. I also think I can massify a bit more or reach those places” (Focus group, participant 1). “It's a very good tool for us so that we don't have to spend an hour with a patient, and we can do more work” (Focus group, participant 11). |
|  |  | *Barriers:*  Barriers they believe could arise when implementing and disseminating this type of resource in our context | *Technical resources and connectivity:*  Especially in the countryside, it was mentioned by professionals as one of the main barriers to implementation, as not the entire population has access to computers or internet connection. | “Connectivity I feel that, for example, I have patients in Santiago del Estero who sometimes do not have the best accessibility too, because I am from Santiago del Estero, I know the place and there are places where the signals are sometimes very complex.... I think that connectivity is one of the biggest barriers” (Focus group, participant 2), “We don't have the tools to see connectivity at home, I am from Mendoza, I don't know if they are all from Buenos Aires, it is very scarce in the population” (Focus group, participant 6). |
|  |  |  | *Cultural barriers:*  The lack of human connection was mentioned as a barrier. Professionals also referred to the strong influence of psychoanalytic tradition in psychotherapy within the country. | “Culturally, we are more about contact and face-to-face contact. I think that this also affects our culture a bit” (Focus group, participant 6). “The patients, we are in a culture very marked by this, of the psychoanalytic and the importance of talking” (Focus group, participant 11). |
|  |  |  | *Therapist beliefs:*  Therapist beliefs or preconceived ideas as barriers, such as the notion that the "personal" aspect of psychotherapy is lost, that online interventions are superficial, or that the individualized, case-by-case approach is compromised. | “In that sense, I feel that perhaps it takes away a bit of the more artisanal part of the therapist modifying the structure of the different protocols for each patient”. (Focus group, participant 2). “You lose the case by case because it's like you're reading what someone else answered, it sounds a bit superficial” (Focus group, participant 13). |
|  |  |  | *Sociodemographic factors (age, education):*  Both age (with older individuals being less inclined to use such interventions) and educational level may act as barriers to access in our context. | “The issue of also the level of education to understand all the instructions well. Maybe that can also be a barrier” (Focus group, participant 8). “Surely there are still older patients where they may not get so confused with the use of an application” (Focus group, participant 2). |
|  | Public sector | Advantages | They mention how implementing these interventions can be useful due to the long waiting lists that currently exist in hospitals, as patients do not receive any treatment during this time. | “I think it would be very useful in the public sector because of the amount of demand, which is impressive in a public hospital, for example, it is impressive” (Focus group, participant 3). “I'm speaking from the public hospital, but I think it could be implemented and I think it happens a lot, that a lot of people arrive without treatment and they can't get it anywhere and the whole system is saturated”. (Focus group, participant 16). |
|  |  | Barriers | Some barriers mentioned may be resistance from professionals, particularly from more senior professionals. | ‘Well, I work in a public hospital, so there is also something about technology that is very difficult for us to implement and that would be great because it would help a lot, but we are a bit alien to it” (Focus group, participant 16). “I also think that public hospital professionals, perhaps not so much as us residents who are generally younger and more up to date, have certain defenses in this regard” (Focus group, participant 16). |
|  | Adherence: | Benefits: | Benefits that these interventions can bring in terms of adherence to such interventions and also in combination with face-to-face treatments, for example by allowing greater use and availability of records. | “The records are great, it makes it much easier to fill in the forms, with boxes so that the person can do it directly from their mobile phone or computer. In that sense it's a great thing” (Focus group, participant 2). “Above all, for patients in fortnightly mode, I think it's a good way to help them keep track” (Focus group, participant 9). |
|  |  | Difficulties: | Professionals mention possible difficulties in terms of treatment adherence. They argue that this type of intervention lacks the commitment of a scheduled appointment with the therapist, which may reduce motivation to engage in treatment. | “It may be more of a procrastination issue, or it may be an encouragement of procrastination” (Focus group, participant 15). “Maybe motivation can go down and you don't want to continue. For example, I think it could be a disadvantage because it is a bit long, so motivation could drop” (Focus group, participant 3). |
|  | Future improvements | | Suggestions and proposals to improve the platform. Professionals mention that, on one hand, it would be necessary to adapt the platform for use on mobile devices. On the other hand, they also suggest improvements in aesthetics, such as adding more colors or more eye-catching buttons. | “It seems to me that some aspects of attractiveness could be improved” (Focus group, participant 7). “I feel that it would be somewhat counterproductive for an application to be more complex from the mobile phone than from the computer, as it is very likely that some patients, particularly the majority, tend to connect from the mobile phone” (Focus group, participant 2). |
